# Early effects of gene duplication on the robustness and phenotypic variability of gene regulatory networks

**DOI:** 10.1186/s12859-022-05067-1

**Published:** 2022-11-28

**Authors:** Yuridia S. Posadas-García, Carlos Espinosa-Soto

**Affiliations:** Instituto de Física, Universidad Autónoma de San Luis Potosí, San Luis Potosí, Mexico

**Keywords:** Gene duplication, Gene regulatory network, Robustness, Phenotypic variability

## Abstract

**Background:**

Research on gene duplication is abundant and comes from a wide range of approaches, from high-throughput analyses and experimental evolution to bioinformatics and theoretical models. Notwithstanding, a consensus is still lacking regarding evolutionary mechanisms involved in evolution through gene duplication as well as the conditions that affect them. We argue that a better understanding of evolution through gene duplication requires considering explicitly that genes do not act in isolation. It demands studying how the perturbation that gene duplication implies percolates through the web of gene interactions. Due to evolution’s contingent nature, the paths that lead to the final fate of duplicates must depend strongly on the early stages of gene duplication, before gene copies have accumulated distinctive changes.

**Methods:**

Here we use a widely-known model of gene regulatory networks to study how gene duplication affects network behavior in early stages. Such networks comprise sets of genes that cross-regulate. They organize gene activity creating the gene expression patterns that give cells their phenotypic properties. We focus on how duplication affects two evolutionarily relevant properties of gene regulatory networks: mitigation of the effect of new mutations and access to new phenotypic variants through mutation.

**Results:**

Among other observations, we find that those networks that are better at maintaining the original phenotype after duplication are usually also better at buffering the effect of single interaction mutations and that duplication tends to enhance further this ability. Moreover, the effect of mutations after duplication depends on both the kind of mutation and genes involved in it. We also found that those phenotypes that had easier access through mutation before duplication had higher chances of remaining accessible through new mutations after duplication.

**Conclusion:**

Our results support that gene duplication often mitigates the impact of new mutations and that this effect is not merely due to changes in the number of genes. The work that we put forward helps to identify conditions under which gene duplication may enhance evolvability and robustness to mutations.

## Background

Biologists have long recognized the evolutionary relevance of gene duplications [1–3]. In fact, a large fraction of genes in a wide range of genomes is easily recognized as the product of gene duplication, especially in the eukaryotic branch of the tree of life [2, 4–6].

Discussion around the evolutionary consequences of gene duplication often focuses on its long term effects and the final fate of duplicate genes [1, 2, 7–10]. Some, such as Ohno in his classic book, defend that duplication creates a redundant gene without early phenotypic consequences [1]. In Ohno’s model of evolution, duplication paves the way for significant adaptive evolution despite the absence of early effects. Others defend a different mode of evolution in which the ancestral gene, before duplication, is multifunctional and, after duplication, random mutation starts disrupting different functions in the two duplicates [11]. In a different scenario [12] most of a gene’s ancestral secondary functions are not carried out at an optimal rate; duplication would be beneficial from the start as it enhances these secondary functions. Later evolution can then improve further such functions by adjusting one of the duplicate genes [12, 13]. The debate on the preponderance of the different scenarios is lively and enriched by insightful theoretical studies and substantial empirical evidence [8–11, 14, 15].

Gene duplication is purportedly associated to the duplication and specialization of anatomical structures [16], evolutionary innovations and adaptive radiations [17, 18], speciation through neutral evolution [19] and buffering of mutations [20]. However, there is not universal acceptance of some of such claims. Regarding evolutionary innovation, numerous studies support that gene duplication plays a significant role [17, 18, 21]. One example concerns experimental evolution of the bacterium *Pseudomonas aeruginosa* [22]. Such study considered evolution in environments with carbon sources that initially produced only marginal growth and in environments with carbon sources that allowed easier growth from the start. In the first case, cell populations that eventually thrive tend to bear mutations in recent duplicates, but not in the second case [22]. This result suggests that modification of duplicates may be especially fruitful when evolution requires to create new functions almost from scratch. Other studies suggest that gene duplication may favor adaptive evolution through changes in gene expression. Analyses of gene expression in species as different as yeast [23, 24], fruit flies [24], mice and human [25] have shown that gene expression diverges more easily for genes with a duplicate than for singleton genes.

There are also warnings against conceding an excessive significance to gene duplication in adaptive evolution [26, 27]. For example, Carroll considers that gene duplication may have paved the way for the evolution of many important traits in vertebrates but, he argues, the fundamental means in the evolution of morphology in both vertebrates and arthropods is the evolution of gene regulation [27]. Moreover, selection can act against fixation of duplicate genes because any additional genetic material may be associated to increased mutational hazards; thus, gene duplicates may be scarce in organisms living in large populations [7, 28]. These observations may help to explain why gene duplication is apparently not prevalent in many prokaryotic species [6, 29, 30].

Gene duplication may also affect evolution through its potential effect on mutational robustness [31]. Mutational robustness is a genotype’s ability to endure random mutations with little or no phenotypic effects [32]. At first sight, robustness may seem to hamper evolution because it diminishes an organism’s opportunity to change phenotypically through mutation. Notwithstanding, populations with mutationally robust organisms are able to contain greater amounts of genetic variation because mutations are not as easily rejected by selection as in populations with less robust organisms. As a result of such a surplus of genetic variation, the population as a whole may explore more phenotypic variants, increasing the chances of finding one that is beneficial [33–36].

The main rationale for how duplication enhances mutational robustness is simple: it creates gene backups, although this is not the only way in which duplication enhances robustness [20, 37, 38]. Evidence supporting an association between duplication and robustness comes from different kinds of studies. Makino and Kawata showed that Drosophila species that live under a wider range of environments have a larger proportion of duplicate genes in their genome [39]. In mammals, the proportion of genes that arose through small-scale duplications is also associated to the species’ habitat variability [40]. Makino and Kawata also found that a species invasiveness, that implies an ability to tolerate exposure to new different environments, is associated to a larger fraction of duplicate genes in a wide range of animal groups [41]. Although, strictly speaking, this evidence associates gene duplication to robustness in the face of environmental perturbations, many other studies have shown that robustness to different kinds of perturbations are often positively associated [42–49].

Notwithstanding, apparently gene duplication does not always lead to increases in robustness. Diss et al. found that although many duplicates can compensate for the loss of interactions after eliminating their paralogues, in many other cases protein-protein interactions of duplicate pairs require the presence of both paralogues [50]. In these latter cases, evolution of gene duplicates has lead to greater fragility instead of greater robustness.

A better understanding of how gene duplication affects adaptive evolution and mutational robustness requires us to take into account that genes do not contribute to the phenotype in an independent manner. Rather, the effect of the duplication of a particular gene depends on the genetic context in which such duplication occurs. The reason is that gene products construct phenotypic traits through their interactions with other gene products. Indeed, Soyer argues that many of the contradictions between theoretical predictions and empirical evidence regarding gene duplication may occur because of not taking into account the complexity of genetic interactions [51]. Therefore, we need to understand how the effect of a gene duplication percolates through the mesh of gene interactions.

Previous research has already addressed how gene duplications affect biological systems and their evolution considering that duplicates are embedded in networks of molecular interactions. Most of this research has focused on the long term effects of gene duplication on biological networks. Many of these studies address how evolution through gene duplication affects the structure of biological networks [52–54]. An interesting example is the work of Soyer that studied signaling networks to show that duplication and a specific selection regime leads to the evolution of independent signaling pathways [55]. Indeed, empirical evidence also supports that gene duplication has significant effects on the structure of biological networks; for example, Teichmann and Babu found that a large fraction of the transcriptional regulatory interactions in yeast appear in paralogues that preserved them from the ancestral gene through duplication [5].

Other researchers interested in the long term effects of gene duplication on biological networks have addressed questions on the dynamics of such networks. That is the case of Aldana et al., who used a Boolean model to analyze the dynamics and evolution of gene regulatory networks; they found that networks that evolve through duplication and mutation of duplicated genes are prone to preserve the ability to produce the same gene activity patterns as the ancestral network [56]. Another illuminating study simulated the evolution of gene regulatory networks under fluctuating environments and found that duplicates are more easily retained when such environmental fluctuations are not predictable [57]. In addition, the authors found that the effect of different kinds of mutations is less severe in networks with more genes due to gene duplication. Other works have simulated the evolution of gene regulatory networks, taking into account gene duplications among other kinds of mutations, to study how the ability to produce beneficial mutations evolves [58], differentiation of subpopulations [59], or the evolution of developmental processes like the production of metamers and their differentiation [60].

Despite the many studies that have addressed the long term consequences of gene duplication on biological networks, there are still many substantial gaps in our understanding of the evolutionary effects of gene duplication. As Kondrashov argues, it is necessary to delve deeper into the organismal and phenotypic effects early after gene duplication to better understand the evolutionary consequences of gene duplication [61]. Few studies have addressed the immediate and short term effects of gene duplication on biological networks. Notwithstanding, they have yielded important insights. For example, that gene duplication is less prone to perturb the phenotype when such duplication encompasses either very few or nearly all the genes in the network [62] or that duplication of proteins in the intermediate layers of a signalling network are more likely to withstand duplication without affecting the phenotype [63]. Indeed, many of the open questions around the role of duplication in adaptive evolution and its interplay with robustness in biological networks concern these early effects: What kind of networks are more prone to endure gene duplication without manifesting phenotypic effects? How does gene duplication affect the sensitivity to new mutations? How similar are the effects of the appearance of a new gene introduced by gene duplication and when the new gene is unrelated to previously existing genes? How does gene duplication affect mutational access to new phenotypes? What kind of phenotypes are more likely to remain accessible through mutation after gene duplication?

Our work focuses on gene regulatory networks (*GRNs*). Such networks comprise sets of genes that regulate each other, for example by controlling transcription [64]. Gene regulatory networks establish the gene expression patterns that a cell displays, thus defining the cell’s properties. They are responsible for the developmental appearance of many traits in all organisms and their evolutionary modification has produced innumerable adaptations and innovations [65, 66]. A cell starts a dynamic trajectory of changes in gene activity from an initial gene activity pattern, established by environmental signals, genes outside the network, morphogenetic signals from other cells or maternal factors. Active genes modify, through regulatory interactions, the activity of other genes in the network, turning some of them off and others on. The new changes in gene activity can lead to new alterations in the expression of other genes, thus initiating a cascade of such changes. Eventually, gene activity settles. Here we consider such a final gene activity pattern as the network’s phenotype. After all, it is the output of a developmental process, the sequence of changes in gene activity, driven by genetically encoded regulatory interactions.

Here we use a simple model of the developmental dynamics of gene regulatory networks to assess the effects of the duplication of single transcription factors on the ability to maintain the network’s original expression pattern and to evolve new such phenotypes through mutation. We find that those networks that are more robust to single-interaction mutations are also more prone to produce the same expression pattern after gene duplication and that it is easier to withstand duplication of one gene than insertion of a new transcription factor unrelated to previously existing ones. Moreover, we found that those networks that preserved the ability to produce the original phenotype after duplication increased further their robustness to mutations. We also found that the effect of mutations after duplication depends on both the kind of mutation and genes involved in it. In addition, we studied how duplication affects the access to new phenotypes through new additional mutations. Our results suggest that those phenotypes that had easier access through mutation before duplication had higher chances of remaining accessible through new mutations after duplication. In sum, our work contributes to the discussion of the role of gene duplication on evolution by throwing light on the conditions under which gene duplication may enhance evolvability and mutational robustness.

## Methods

### Model

Different kinds of models have allowed the study of GRNs, including thermodynamic models that allow assessing gene expression under equilibrium conditions [67, 68], partial differential equation models [69], modifications of the Gillespie algorithm to consider stochastic reactions [70], or Boolean networks [71]. We used a slight variation of a model first proposed by A. Wagner to study the dynamics and evolution of gene regulatory networks [62]. Despite its level of abstraction, this model has shown its convenience to address topics on the evolution of GRNs as diverse as the role in evolution of plasticity [72–74] and gene duplication [62] or the evolution of sensitivity to mutations [46, 75–80], epistasis [81, 82] organismal complexity [83], modularity [80, 84, 85] or hybrid incompatibility [86, 87].

The main difference of the model that we use with respect to Wagner’s original proposal is that our networks contain two different kinds of genes: transcription factor and structural genes. Transcription factor genes regulate the activity of other genes, be them structural, themselves or other transcription factor genes. Structural genes do not regulate the activity of other genes; we consider that their activity defines a cell’s phenotypic properties. In our design, duplication only affects transcription factor genes. That is, the number of structural genes remains the same after gene duplication. Because we characterize phenotypes only in terms of structural genes, the size of phenotype space remains invariant, enabling fairer comparisons of phenotypic effects of perturbations before and after gene duplication. In our setup, the number of transcription factor genes (before duplication) equals $N_r=12$ and the number of structural genes equals $N_s=6$.

A matrix ${\textbf{M}}$ of $N_r + N_s$ rows and $N_r$ columns stores information on a GRN’s genetically-defined regulatory interactions. Its real-valued entries $m_{i,j}$ indicate the regulation that gene *j* exerts on gene *i*. The magnitude of $m_{i,j}$ specifies the intensity of the regulatory interaction and its sign indicates whether its effect is activatory (positive) or inhibitory (negative).

At any time point *t*, we describe the network’s activity state using a vector ${\textbf{s}}^t$ of size $N_r+N_s$. A positive or negative entry $s_i^t$ indicates that, at time *t*, the *i*-th gene is active or inactive, respectively. Network dynamics start from a predefined initial condition ${\textbf{s}}^0$. In nature, extracellular signals, environmental inputs, maternal factors or genes external to the network define such an initial state. Then, gene activity changes, updating the system state, according to:

1 $$\begin{aligned} \begin{aligned} s_i^{t+1} = \sigma _i \left[ \sum _{j=1}^{N_r}m_{i,j}s_j^t\right] \end{aligned} \end{aligned}$$

where $\sigma _i(x)$ denotes the step function:

2 $$\begin{aligned} \begin{aligned} \sigma _i(x)= {\left\{ \begin{array}{ll} 1\text {,} &{}\text {if } x>0\\ s_i^t\text {,} &{}\text {if } x= 0\\ -1\text {,}&{}\text {if } x < 0 \end{array}\right. } \end{aligned} \end{aligned}$$

Equation 1 implies that a gene’s new activity state depends on the sign and magnitude of the regulatory interactions that impinge on it and on the activity state of its regulators. Gene activity changes through a series of time steps until, eventually, the system attains a combination of activity states that it has reached before in that trajectory. That is, there is a system state ${\textbf{s}}^{\tau +\kappa }$ such that ${\textbf{s}}^{\tau +\kappa }={\textbf{s}}^{\tau }$. This occurs necessarily because, in the model, the number of possible system states is finite. Notwithstanding, it usually takes a few time steps to reach such a state. Because the system is deterministic, the system remains locked, following indefinitely the sequence of $\kappa$ system states from ${\textbf{s}}^{\tau }$ to ${\textbf{s}}^{\tau +\kappa -1}$. In dynamic systems parlance, this sequence is an attractor; it may be a fixed point ($\kappa =1$) or a $\kappa$-period limit cycle ($\kappa > 1$). To characterize a GRN’s phenotype we only consider the activity states of the structural genes in a GRN’s attractor. Specifically, a GRN’s phenotype is the indefinitely repeating sequence of structural gene activity patterns in the GRN’s attractor. Consequently, two different GRNs may produce two different attractors but still produce the same phenotype if they lead to the same dynamic pattern for structural genes.

### Mutation, duplication and gene addition

Mutation implies, in our model, either the addition, deletion or modification of a regulatory interaction. We represent mutations as changes in the matrix of genetically-encoded regulatory interactions ${\textbf{M}}$. We add a new regulatory interaction from gene *j* to gene *i* by changing the value of entry $m_{i,j}$ from 0 to a non-zero value. In this case, we pick the new value randomly from the standard normal distribution. We delete the regulation upon gene *i* by gene *j* by changing to zero the value of a non-zero entry $m_{i,j}$. To modify the strength of a regulatory interaction, we replace a non-zero entry $m_{i,j}$ with a new value randomly taken from the standard normal distribution but forcing to preserve the sign of the original value of $m_{i,j}$.

Gene duplication adds a copy of a preexisting transcription factor-encoding gene. In the model, we bring duplication of gene *i* to fruition by adding a new column and a new row into the matrix ${\textbf{M}}$. We insert the new column on position $N_r+1$ and the new row on position $N_r+1$ and they are identical to the *i*-th column and row, respectively. The new entry $m_{N_r+1,N_r+1}$ copies its value from entry $m_{i,i}$. The vector ${\textbf{s}}$, that describes the system state, also increases its length one entry. The two gene copies have the same activity state in the new default initial condition that the original gene had in the predefined initial condition before duplication.

We also considered two kinds of addition of non-duplicate genes to differentiate between the effects of duplication and those of adding a gene without any relationship to preexisting genes. The first such kind of gene addition consisted in attaching a new transcription factor gene with as many interactions as the average number of interactions per gene in the network. The second one implied adding a transcription factor gene with the same number of interactions as the duplicated gene in the corresponding experiment. In both cases, the interactions of the new gene were connected randomly to other genes in the network. The weights of the new interactions were picked randomly from the standard normal distribution. We found that both kinds of gene addition produced very similar results. Thus, we present here only results for the first one.

### Robustness and similarity

A GRN starts its dynamics from a predefined initial condition ${\textbf{s}}^0$ and eventually reaches an attractor, as described above. We define a GRN’s phenotype as the indefinitely repeating sequence of structural gene activity patterns in the GRN’s attractor. We measured the capacity of GRNs to buffer different kinds of perturbation: single-interaction mutations, gene duplication and non-duplicate gene addition. Robustness to perturbations of class *K* ($R_K$) is the fraction of such perturbations that preserve unaltered a GRN’s original phenotype. It tells us how often a certain kind of perturbation produces no phenotypic effect. We also wanted to evaluate the degree of phenotypic divergence that perturbations produce. We thus define the similarity between two phenotypes as the fraction of structural genes with the same state (either active or inactive) in the two phenotypes [85]. Additional file 1 illustrates how we measure this pairwise similarity when gene activity in neither phenotype fluctuates (panel a) and when there are fluctuations in gene activity (panel b). We refer to the average similarity between the original phenotype and those produced by a set of perturbations of class *K* as the similarity after perturbation *K* ($S_K$).

To assess a GRN’s mutational robustness ($R_\mu$) and its similarity after mutations ($S_\mu$), we create $2(N_r+N_s)N_r$ single-mutants of that network, each with an independent random mutation. For each such mutation, we first pick at random an entry in ${\textbf{M}}$. The kind of mutation that we apply depends on the value of the entry that we picked. The reason is that, given that entry, not every mutation is possible; for example, we cannot delete an interaction that does not exist. For non-zero entries, deletion or modification of an interaction occurs with the same probability.

We evaluated a GRN’s ability to buffer duplications, as indicated by $R_D$ and $S_D$, by duplicating each of the $N_r$ transcription factor genes, one at a time, and finding the phenotype that such perturbation yields. In the case of addition of non-duplicate genes, we add $N_r$ random transcription factor genes, as explained above, and thus calculate $R_a$ and $S_a$.

We also studied the effect that duplication and non-duplicate gene addition had on $R_\mu$ and $S_\mu$. For those analyses, we either added a new random regulator gene or duplicated a single random transcription factor gene in each network and then we assayed $R_\mu$ and $S_\mu$.

### Random sample of GRNs

Previous research has shown that networks that produce the same phenotype *A* comprise huge sets of networks that can be traversed through single mutation steps without ever losing the ability to yield *A* [46]. We generated an initial sample $X_i$ of random networks that produce the same phenotype *A* through a Monte Carlo walk as described in [46], which guarantees a uniform probability distribution for picking a GRN in the set [46, 88].

As a starting point in the walk we use a network that, by design, yields phenotype *A* when its dynamics start from ${\textbf{s}}^0$. Then, we start a Monte Carlo walk in which each step implies mutating randomly an interaction. The mutation is accepted only if the new mutated network is also able to yield *A* when its dynamics start from the predefined initial condition ${\textbf{s}}^0$. Otherwise, the walk goes back to its previous position. We include in our sample a network after every $20(N_r+N_s)N_r$ mutational steps. Thus, any similarity between consecutively sampled networks is due to chance and not inherited. Along the walk, we force networks to have a number of interactions [C] in the interval [C-2,C+2] so that all networks in our sample have a very similar number of connections, thus allowing fairer comparisons. In our setup, $C=(N_r+N_s)N_r/4$, which is similar to the connectivity of GRNs sustained on empirical evidence [85, 89].

Along this research, we study two additional sets of networks, besides $X_i$: i) the set $X_{D}$ of networks with a duplicated gene, that we obtain by duplicating a random transcription factor gene in each network in $X_i$; and ii) the set $X_{a}$ of networks with an additional non-duplicate gene, that we obtain by inserting a new random transcription factor gene in each network in $X_i$. We thus perform paired comparisons between $X_i$ and $X_{D}$, $X_i$ and $X_{a}$, and $X_{D}$ and $X_{a}$.

The code for simulations and analyses is written in C++ and we performed all statistical analyses in R, using $\alpha =0.01$. All the data that we used was generated using our code available at https://github.com/cespinosas/Duplication2022.

## Results

### Robustness and similarity

**Fig. 1:**
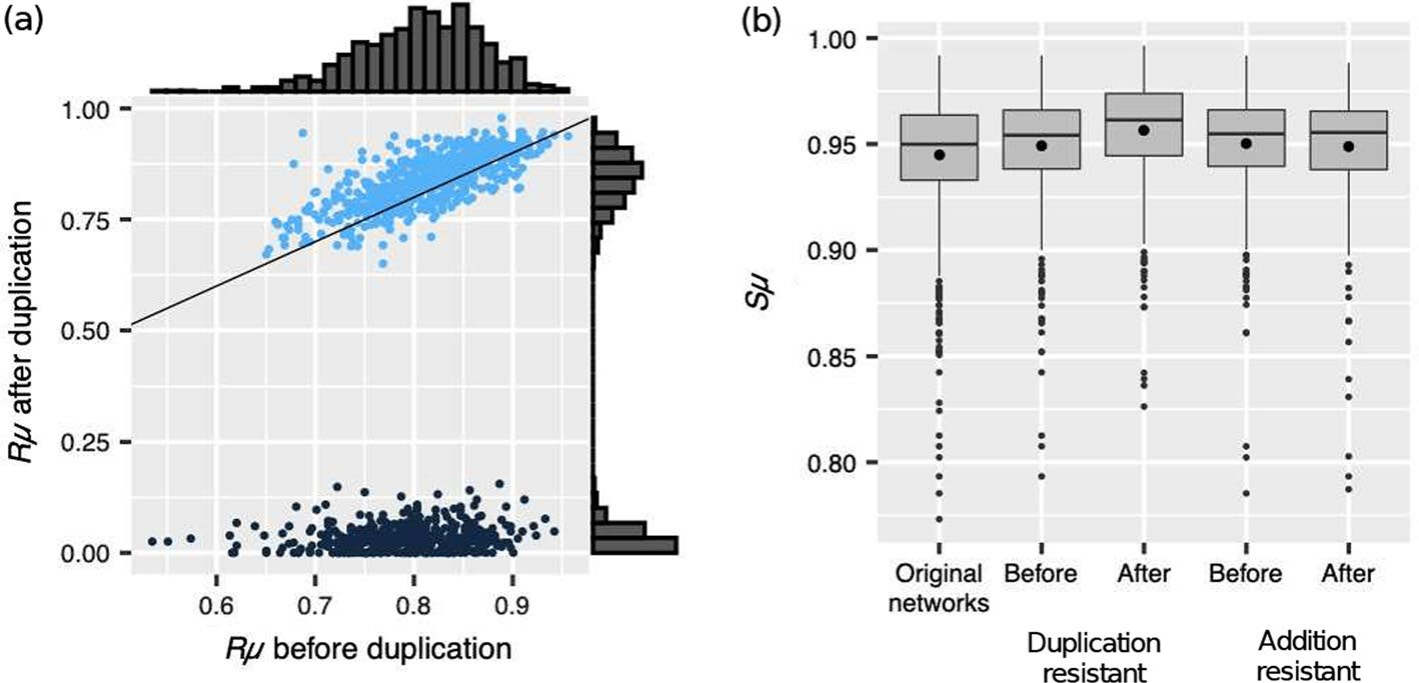
Mutational robustness and phenotypic similarity after mutations before and after gene duplication. **a** Mutational robustness before and after duplication. Each dot represents a GRN in our random sample; its position along the horizontal and vertical axes reflects the network’s $R_\mu$ before and after gene duplication, respectively. Light blue points represent duplication-resistant GRNs and the dark ones duplication-susceptible GRNs. The continuous line is the identity line; points above and below such line indicate, respectively, GRNs that increased or decreased $R_\mu$ after duplication. **b** Phenotypic similarity after mutations. The similarity to the original phenotype of the phenotypes that mutation produces is significantly greater after adding a duplicate gene in duplication-resistant GRNs but not after adding a non-duplicate regulator gene in addition-resistant GRNs. The panel includes data for 1,000 GRNs before adding a new gene, 588 duplication-resistant GRNs and 413 addition-resistant GRNs. We note that duplication-resistance and addition-resistance are not mutually exclusive categories

We study a random sample $X_i$ of $10^3$ GRNs that produce the same phenotype *A*. We refer to the fraction of mutations that do not alter the phenotype of a GRN as that network’s mutational robustness ($R_\mu$). The GRNs in our random sample $X_i$ were highly robust against mutations (mean $R_\mu \pm$SD: $0.81\pm 0.06$) (Fig. 1a). We also evaluated similarity after mutations ($S_\mu$), which refers to the average resemblance of phenotype *A*, produced by all networks in sample $X_i$, to the phenotypes that each network in $X_i$ produces after subjecting it to mutations (see details in Methods and Additional file 1). Mean $S_\mu$ of the networks in sample $X_i$ was $0.94\pm 0.028$ (Fig. 1b). As expected, there is a strong positive correlation between $R_\mu$ and $S_\mu$ (Spearman’s $\rho =0.82$, $p=5.2\times 10^{-240}$). Throughout this paper, this correlation is also found every time we evaluate $R_\mu$ and $S_\mu$.

We also inquired about the phenotypic effects that gene duplication has on GRNs in our sample $X_i$. Analogously as for mutations, we define $R_D$ as the fraction of all the $N_r$ different duplications of a single regulation gene that preserves phenotype *A*. We also define $S_D$ as the average resemblance between a network’s original phenotype *A* and the ones that duplication of transcription factors produces (see Methods and Additional file 1). Among the networks in sample $X_i$, mean $R_D$ equals $0.60\pm 0.17$ and mean $S_D$ equals $0.88\pm 0.08$. Again, as expected, $R_D$ and $S_D$ strongly correlate ($\rho =0.82$, $p=9.0\times 10^{-241}$).

To discern between effects exclusively due to duplication and effects that result from merely adding a new regulator, we considered two kinds of non-duplicate gene addition. In one of them, we added a new regulator gene with as many interactions as the average for the whole network. On the other kind of non-duplicate gene addition, the new regulator gene had the same number of connections as a randomly picked regulator gene in the network. In both cases, we wired randomly the interactions of the new gene to the rest of the network genes. Here we only delve into the first kind of gene addition. Notwithstanding, our observations are quite similar in both cases. We found that, in networks in sample $X_i$, the fraction of independent single-gene additions that did not change the phenotype, $R_a$, was $0.43\pm 0.19$. The mean similarity of the phenotypes after non-duplicate gene addition to a network’s original phenotype, $S_a$, was $0.81\pm 0.09$. Again, there was a strong positive correlation between $R_{a}$ and $S_{a}$ ($\rho =0.75$, $p=1.6\times 10^{-181}$). This data shows that the GRNs were more capable to buffer the addition of a duplicate gene than the addition of a non-duplicate gene. $R_D$ was significantly greater than $R_a$ (Mann-Whitney U test; $U=348,252$, $p=1.1\times 10^{-88}$) and $S_D$ than $S_a$ ($U=439,061$, $p=4.1\times 10^{-99}$).

Previous research suggests that networks robust to one kind of disturbance (i.e., against mutations) are often also robust to others such as recombination [82] or to transient perturbations due to developmental noise or environmental fluctuations [46]. Is this observation also valid for gene duplications? This is indeed the case. We found a strong positive correlation between $R_\mu$ and $R_D$ ($\rho =0.66$, $p=4.7\times 10^{-127}$) and between $S_\mu$ and $S_D$ ($\rho =0.72$, $p=2.5\times 10^{-158}$). The observation is also true for the addition of new non-duplicate genes, albeit the correlation is not as strong in this case. $R_\mu$ and $R_a$ had a positive correlation ($\rho =0.44$, $p=8.9\times 10^{-48}$) just as similar as $S_\mu$ and $S_a$ ($\rho =0.44$, $p=7.8\times 10^{-49}$).

Next, we analyzed the effect of mutations after adding a gene through duplication. For each network in $X_i$ we picked at random one transcription factor gene and duplicated it. We thus created a new set of GRNs, $X_D$, that we can compare to GRNs in $X_i$ to assess the effect of gene duplication. Next, we perturbed GRNs in $X_{D}$ with mutations to detect changes in their $R_\mu$ and $S_\mu$ behavior after duplication with respect to the phenotype *A* that GRNs in $X_i$ produced before duplication. After duplication, $R_\mu$ decreased significantly to $0.51\pm 0.4$ (Wilcoxon signed-rank test; $W=354,908$, $p=7.5\times 10^{-37}$), and $S_\mu$ to $0.85\pm 0.17$ ($W=366,543$, $p=2.0\times 10^{-37}$).

The distribution of $R_\mu$ after gene duplication, in $X_{D}$, is bimodal (Fig. 1a). This suggests the existence of two different classes of networks that respond differently to gene duplication. We thus inquired whether the ability to produce phenotype *A* after gene duplication explained the two different kinds of response to mutations. Most ($58.8\%$) of the GRNs in $X_D$ still produced phenotype *A* after duplication of a regulator gene picked at random. Hereafter we refer to such networks as *duplication-resistant GRNs*. Within duplication-resistant GRNs, the fraction of mutations that did not alter the phenotype of the GRNs in $X_D$ was $R_\mu =0.841\pm 0.059$, and the similarity of their phenotypes to the original phenotype *A* was $S_\mu =0.957\pm 0.024$ (Fig. 1b). That is, duplication produced in these networks a significant increase in $R_\mu$ from $0.823\pm 0.057$ to $0.841\pm 0.059$ ($W=129,712$, $p=6.6\times 10^{-34}$; Fig. 1a) and $S_\mu$ from $0.949\pm 0.026$ to $0.957\pm 0.024$ ($W=133,589$, $p=2.0\times 10^{-30}$, Fig. 1b). Such increases may seem small, however, they imply that, on average, duplication reduces the effects of mutation in around $10\%$ for $R_\mu$ and $16\%$ for $S_\mu$. In contrast, mutations after a gene duplication that does not yield the original phenotype *A* are only rarely able to recover such original phenotype (Fig. 1a). In such *duplication-susceptible GRNs* we found a significant decrement in $R_\mu$ from $0.79\pm 0.06$ to $0.04\pm 0.03$ ($W=85,078$, $p=1.5\times 10^{-69}$) and in $S_\mu$ from $0.94\pm 0.03$ to $0.69\pm 0.17$ ($W=85,078$, $p=1.5\times 10^{-69}$).

Yet, duplication-susceptible GRNs displayed high similarity to the new phenotype that duplication created ($0.95\pm 0.03$). Nonetheless, our focus is on a GRN’s ability to preserve the original pre-duplication phenotype.

We also studied the effect of adding a non-duplicate gene on $R_\mu$ and $S_\mu$. Thus, for each GRN in sample $X_i$ we added a new random transcription factor gene, as detailed in Methods, to obtain a new set of GRNs that we call $X_a$. In this case, $R_\mu$ equals $0.35\pm 0.4$ and $S_\mu$ equals $0.76\pm 0.2$, which means a decrease in $R_\mu$ ($W=453,778$, $p=4.6\times 10^{-119}$) and $S_\mu$ ($W=461,009$, $p=4.6\times 10^{-118}$) after non-duplicate gene addition. The decrement in $R_\mu$ and $S_\mu$ after non-duplicate gene addition was more pronounced than after gene duplication ($U=650,574$, $p=9.3\times 10^{-32}$ and $U=634,346$, $p=1.2\times 10^{-25}$, respectively). While almost $60\%$ of the GRNs produced the original phenotype after duplication, only $41.3\%$ of the GRNs conserved the original phenotype *A* after adding a new non-duplicate gene. We refer to such networks as ‘addition-resistant GRNs’.

We also found a bimodal distribution in $R_\mu$ after non-duplicate addition in networks in $X_{a}$. Among addition-resistant GRNs, we did not find a significant difference in $R_\mu$ after non-duplicate gene addition ($R_\mu =0.82\pm 0.06$; $W=36,767$, $p=0.09$), nor in $S_\mu$ ($0.95\pm 0.03$; $W=46,155$, $p=0.16$; Fig. 1b). In contrast, in addition-susceptible GRNs, those networks in which addition of a non-duplicate gene produced a phenotype other than *A*, $R_\mu$ significantly decreased from $0.80\pm 0.06$ to $0.02\pm 0.03$ ($W=172,578$, $p=4.0\times 10^{-98}$) and $S_\mu$ from $0.94\pm 0.03$ to $0.64\pm 0.18$ ($W=172,578$, $p=4.0\times 10^{-98}$). Withal, while mutations in addition-susceptible networks yielded phenotypes that were very different to phenotype *A*, mutations after gene-addition produced a phenotype similar to the one that gene addition alone produced in those networks ($0.94\pm 0.03$).

**Fig. 2:**
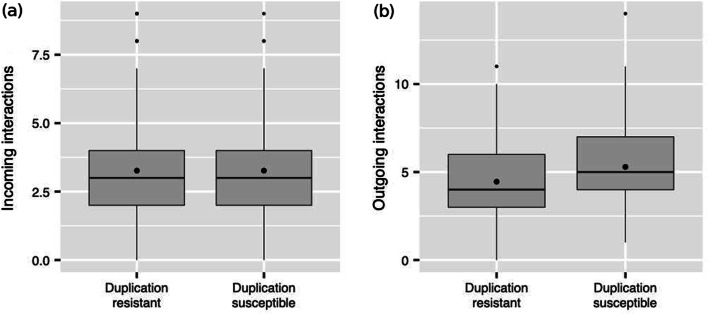
Duplicate gene number of interactions. Number of a duplicate gene’s **a** incoming and **b** outgoing interactions in duplication-resistant and duplication-susceptible GRNs

Are there any structural traits in a duplicate that are associated to the distinction between duplication-resistant and duplication-susceptible GRNs? We measured three properties of the duplicate gene in both kinds of networks: (i) the path length from the duplicate to the nearest structural gene; (ii) the number of the duplicate’s incoming interactions (Fig. 2a); (iii) the number of the duplicate’s outgoing interactions (Fig. 2b). Neither path length (duplication-resistant: $1.17\pm 0.37$; duplication-susceptible: $1.17\pm 0.38$; $U=120,426$; Bonferroni-corrected $p>0.95$) nor the number of incoming interactions (duplication-resistant: $3.26\pm 1.74$; duplication-susceptible: $3.26\pm 1.63$; $W=121,102$; Bonferroni-corrected $p>0.95$) yielded significant differences. In contrast, we found a statistically significant difference ($U=90,842$, Bonferroni-corrected $p=2.7\times 10^{-11}$) in the number of the duplicate’s outgoing interactions between duplication-resistant ($4.45 \pm 5.29$) and duplication-susceptible ($5.2 \pm 1.8$) GRNs. Our results thus suggest that duplication is more likely to preserve the original phenotype when the duplicate gene regulates few other genes.

### Phenotypic similarity after different kinds of mutations

So far, we have analyzed the effects of single-interaction mutations without distinguishing between distinct kinds of such genetic perturbations. However, separate classes of mutations may produce different kinds of phenotypic effects. Thus, we now consider separately the two kinds of mutations that can generate the most contrasting effects, namely, those mutations that (i) delete an interaction or (ii) yield a new interaction. The first experiment consists in the deletion of every interaction in the network, one at a time. In the second experiment, we try 10 positive and 10 negative interactions for every possible missing interaction, one at a time. We extract interaction weights from a standard normal distribution upon which we force the sign that we require. We also consider whether, in the affected interaction, the regulator gene is a duplicate or a singleton and if the target gene is a duplicate or singleton transcription factor or a structural gene (descriptive statistics appear in Additional file 2). We found that the effect of mutations after duplication depends on both the kind of mutation and genes involved.

For each of the two different mutation assays, we used a type II two-way ANOVA to study the effect that the kind of target and regulator have on phenotypic similarity after the two different kinds of mutations (Additional file 3).

**Fig. 3:**
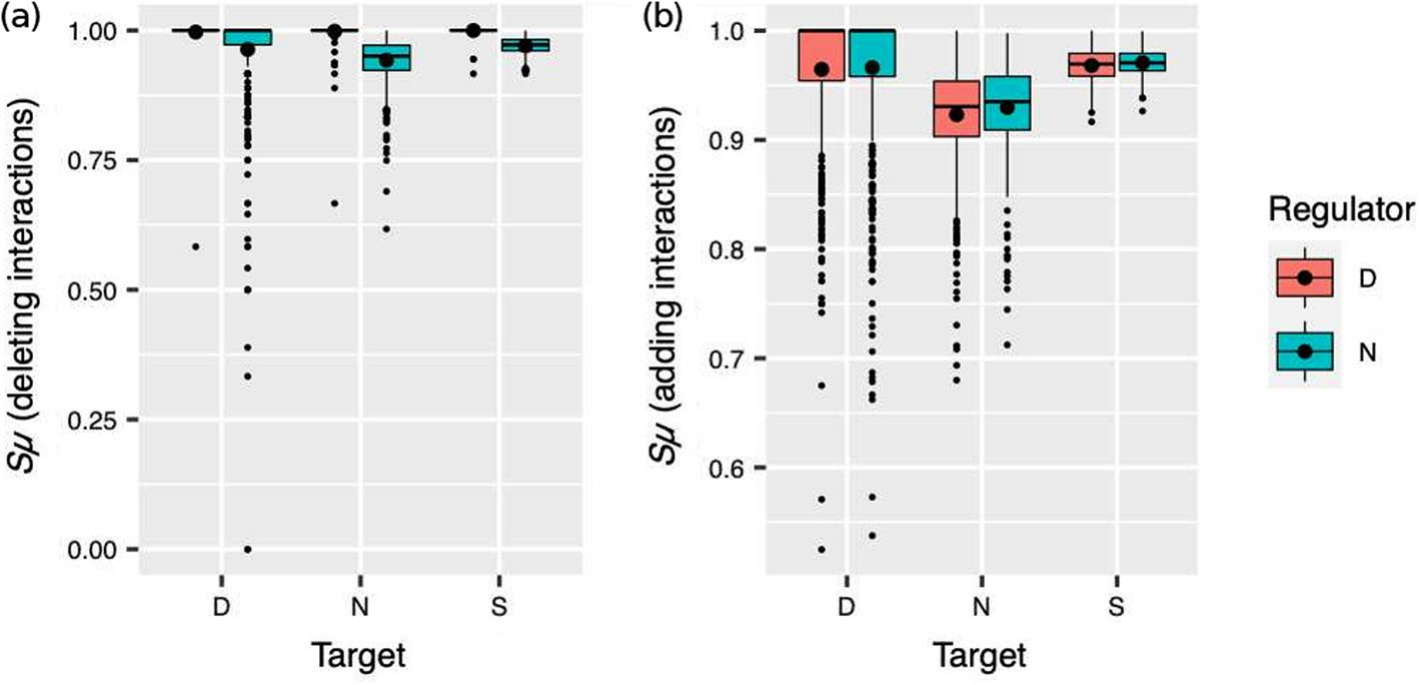
Phenotypic similarity by kind of mutation. Similarity to the original phenotype of the phenotypes produced by GRNs with a duplicate after mutations that **a** delete or **b** add a regulatory interaction. The plots group mutated interactions according to their target gene (duplicate [D], non-duplicate [N] or structural gene [S]) and their regulator gene (duplicate or non-duplicate gene in orange or green, respectively). Black dots indicate the mean $S_\mu$ for every boxplot distribution

When we deleted an interaction, we found statistical differences for regulator and target genes and for their combined effects (Fig. 3a). This means that the kind of gene at the beginning and at the ending point of the perturbed interaction influences the outcome. Notwithstanding, we also found that the effect size for the regulator ($\eta ^2 = 0.13$) was much greater than that for the target gene or for their combined effect ($\eta ^2 = 0.017$ and 0.012, respectively). Moreover, Fig. 3a shows that the kind of regulator gene in the deleted interaction is more relevant than the kind of target gene. Specifically, we found that those interactions with a duplicate as a regulator are better avoiding the effects of mutations that remove interactions.

The pattern was different when we subjected the GRNs to mutations that added a regulatory interaction to the network (Additional file 3; Fig. 3b). In this case, we only found significant differences for the target gene factor, along with a strong effect size ($\eta ^2 = 0.163$). Thus, when we add regulatory interactions, target genes are much more important than regulator genes. What kind of target gene favors a greater $S_\mu$? Clearly, we appreciate in Fig. 3b that those networks in which the target gene in the new interaction is a duplicate tend to produce phenotypes more similar to the original one.

### Gene duplication and access to new phenotypes through mutation

**Fig. 4:**
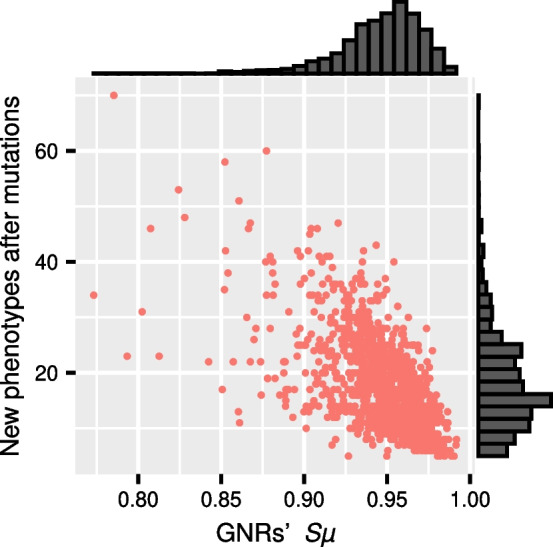
Phenotypic similarity and variability. Relationship between similarity to the original phenotype of phenotypes produced by mutation and the number of distinct new accessible phenotypes in GRNs after duplication. Spearman’s $\rho =-0.59$; $p =1.2\times 10^{-93}$

Next we turned to study how transcription factor duplication affects a GRN’s capacity to generate new distinct phenotypes after mutations or, in other words, the capacity to uncover phenotypes other than *A*. We thus evaluate mutational access to new phenotypes as the number of distinct phenotypes that a GRN yields after perturbing it with $2N_r(N_r+N_s)$ independent random mutations, one at a time. Even when such mutational access to new phenotypes is strongly negatively correlated to the GRNs’ capacity to keep the original phenotype *A* ($S_\mu$, $\rho =-0.59$, $p=1.2\times 10^{-93}$), variation on $S_\mu$ only explains $34.8\%$ of the variation on the number of mutation-accessible phenotypes (Fig. 4).

Originally, GRNs in our initial sample $X_i$ had mutational access to a mean number of $18.33\pm 8.56$ different phenotypes. After duplication, the duplication-resistant GRNs in $X_D$ significantly decreased their number of mutational-accessible phenotypes from $17.78\pm 8.21$ to $15.68\pm 7.90$ ($W=101,092$, $p=2\times 10^{-16}$). In contrast, duplication-susceptible GRNs significantly increased their accessible phenotypes from $19.12\pm 9.47$ to $22.76\pm 15.27$ ($W=30,350$, $p=0.0002$).

GRNs in $X_a$, those with a non-duplicate additional gene, had a different response: they always increased their number of accessible phenotypes. Addition-resistant GRNs increased their access to new distinct phenotypes from $17.06\pm 8.40$ to $21.15\pm 8.58$ ($W=47,464$, $p=0.0002$), and addition-susceptible GRNs from $19.22\pm 8.92$ to $34.55\pm 20.74$ ($W=153,199$, $p=7.5\times 10^{-70}$).

**Fig. 5:**
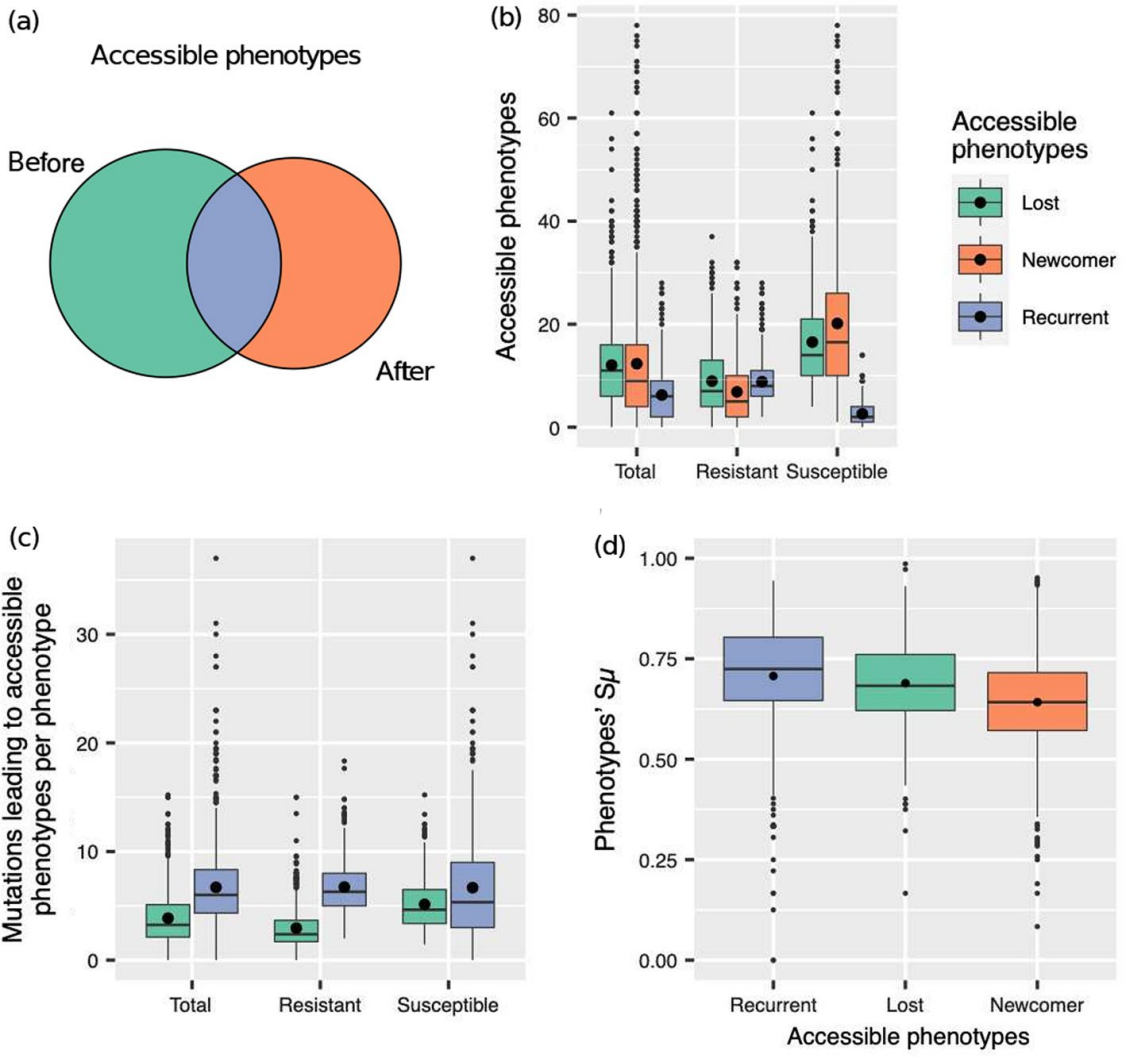
Access to new phenotypes through mutation before and after adding a duplicate gene. **a** New phenotypes accessible through mutation before and after gene duplication. The circle on the left represents the set of phenotypes that a GRN can access before duplication. The one on the right represents the same set but after gene duplication. The green area represents *lost accessible* phenotypes, only accessible before duplication. The orange area represents *newcomer accessible* phenotypes that only become accessible after duplication. The intersection, in blue, represents *recurrently accessible* phenotypes, accessible both before and after gene duplication. **b** The number of distinct accessible phenotypes before and after duplication in all, duplication-resistant and duplication susceptible GRNs. **c** Average number of mutations that lead to each lost and recurrently accessible phenotype. **d** Average similarity of accessible phenotypes to the original phenotype *A*

We detected that some phenotypes that were accessible through mutations before adding a gene were also accessible after adding a duplicate or non-duplicate regulator gene. We called this kind of phenotype ‘recurrently accessible’ (Fig. 5a). We called the new phenotypes that were no longer accessible through mutation after adding a gene the ‘lost accessible’ phenotypes; and those phenotypes only accessible after adding a gene the ‘newcomer accessible’ ones (Fig. 5a).

Most of the new phenotypes produced by mutation in duplication-resistant GRNs in set $X_D$ were recurrent phenotypes ($61.67\%\pm 22.4\%$). In comparison, the proportion of recurrently accessible phenotypes in duplication-susceptible GRNs represented no more than the $14.04\%\pm 12.1\%$ of their whole repertory of mutation-accessible phenotypes.

Otherwise, GRNs in $X_a$, those with a newly added non-duplicate gene, also expressed recurrent phenotypes. Here, this kind of phenotype represented less than the half of the phenotypes produced by the addition-resistant GRNs ($46.29\%\pm 19.92\%$); for addition-susceptible GRNs, the amount decreased further to $8.32\%\pm 7.14\%$.

As we observe in Fig. 5b, duplication-resistant GRNs in $X_D$ lost their access to a mean of $8.95\pm 6.86$ phenotypes after duplication, yet they got $6.86\pm 5.95$ newcomer accessible phenotypes; while, $8.83\pm 4.41$ phenotypes remained accessible on average. In contrast, the average number of recurrently mutation-accessible phenotypes in duplication-susceptible GRNs was $2.60\pm 2.18$. Duplication-resistant GRNs produce a number of recurrently accessible phenotypes that is statistically indistinguishable from the number of lost accessible phenotypes ($W=82,902$, $p=0.36$), but significantly higher than the number of newcomer accessible phenotypes ($W=109,742$, $p=7\times 10^{-15}$). Moreover, they produce significantly more recurrent phenotypes than duplication-susceptible GRNs ($U=225,540$, $p=5\times 10^{-120}$).

In contradistinction to duplication-resistant GRNs, addition-resistant GRNs had more lost accessible phenotypes on average ($16.87\pm 8.35$) and produced more newcomer phenotypes through mutations ($11.96\pm 7.49$; $W=7,877.5$, $p=7\times 10^{-44}$) than recurrent phenotypes ($9.18\pm 4.58$; $W=26,528$, $p=9\times 10^{-8}$).

What kind of phenotypes are more likely to remain accessible through mutation after duplication? We speculated that phenotypes more similar to the original phenotype were more likely to be recurrently-accessible. Across all networks in our sample, the value of $S_\mu$ after duplication for a typical recurrently accessible phenotype averages $0.71\pm 0.12$, that of lost accessible phenotypes $0.69\pm 0.1$ and the one of newcomer accessible phenotypes $0.64\pm 0.12$ (Fig. 5d). Indeed, recurrently accessible phenotypes are significantly more similar to the original phenotype *A* than the other classes of phenotypes (lost: $W=258,458$, $p=2\times 10^{-7}$ and newcomer: $W=301,904$, $p=1\times 10^{-36}$). We notice the same trend, although slightly more pronounced, when we focus exclusively on duplication-resistant GRNs. In such duplication-resistant GRNs, average $S_\mu$ is higher ($0.75\pm 0.08$) for recurrently accessible phenotypes than for lost accessible phenotypes ($0.67\pm 0.11$; $W=130,705$, $p=1\times 10^{-39}$) and for newcomer accessible phenotypes ($0.67\pm 0.12$; $W=117,014$, $p=9\times 10^{-37}$).

We also revised the different kinds of phenotypes in GRNs after addition of a non-duplicate gene. In this case, we observe a similar pattern as for GRNs after duplication. For all the networks in our sample, the mean value of $S_\mu$ after addition of a non-duplicate is $0.71\pm 0.14$ for recurrently accessible phenotypes, $0.69\pm 0.1$ ($W=239,074$, $p=3\times 10^{-8}$) for lost accessible phenotypes and $0.61\pm 0.11$ ($W=334,184$, $p=3\times 10^{-61}$) for newcomer accessible phenotypes.

We also hypothesized that perhaps those phenotypes that had easier access through mutation before duplication were more likely to remain mutation-accessible after duplication. To assess this possibility we counted the number of different mutations leading to each distinct new phenotype before duplication. Figure 5c shows that, indeed, the average number of mutations leading to each recurrently-accessible phenotype ($7.03\pm 3.93$) is greater ($W= 379,310$, $p=5\times 10^{-91}$) than the number of mutations leading to lost accessible phenotypes ($3.95\pm 2.33$). The difference holds when we only take into account duplication-resistant GRNs ($6.73\pm 2.42$ and $3.07\pm 1.94$; $W=153,032$, $p=4\times 10^{-82}$). Even though there are fewer recurrently-accessible phenotypes in duplication-susceptible GRNs than in duplication-resistant GRNs (Fig. 5b), the number of mutations leading to each such recurrently-accessible phenotypes in both classes of networks is statistically indistinguishable (duplication-susceptible GRNs: $7.52\pm 5.52$; $U=113,052$, $p=0.19$; Fig. 5c). Our observations support that phenotypes with easier mutational access before duplication are more likely to remain accessible through mutations after duplication. It is noteworthy that this effect also appears in addition-resistant GRNs. In this case, there are more mutations leading to each recurrently accessible phenotype ($6.47\pm 2.23$) than to each lost accessible phenotype ($3.03\pm 2.10$; $W=73,816$, $p=1\times 10^{-58}$).

## Discussion

Gene duplication is an important phenomenon on evolution [1, 3]. Notwithstanding, it is not equally pervasive in all kinds of organisms, genes and evolutionary scenarios [4, 6, 7, 22, 27, 29, 30, 90, 91]. Therefore, it is necessary to understand what are the conditions that promote the appearance and fixation of new duplicates. In this endeavor, we require to consider how the perturbation that gene duplication implies percolates through the web of gene interactions. To advance on this front we studied the early effects of gene duplication in a simple widely-known model of the developmental dynamics of gene regulatory networks.

Our analyses identified patterns in the relationship between duplication and the production of variation. Although *de novo* birth of new genes [92, 93] and horizontal transfer [94] cannot be neglected, the role of gene duplication in expansion of the number of genes is clearly prevalent in eukaryotes, the organisms with more genes in their genome [2]. In our study of gene regulatory networks with a new additional regulator gene we found that it is much more likely to preserve the original phenotype if the new gene is a copy of a previously existing one than if it is unrelated to any other gene in the network. That is, growth through gene duplication implies less chances of significant perturbation on a GRN’s gene activity pattern. This observation may help to explain the prevalence of gene duplication in eukaryotes. Perhaps contraintuitively, it may also be relevant for the emergence of new genes in prokaryotes through horizontal gene transfer. The reason would be that genes acquired through horizontal transfer are usually not entirely new genes but homologues of other genes in the host genome [29, 94]. Indeed, they are often misidentified as the product of gene duplication [29].

Previous research suggests that robustness to different kinds of genetic and non-genetic perturbations is often positively correlated [44, 45]. This applies to systems and processes as different as RNA folding [43, 95], development of morphological traits [42, 96, 97], fitness components [48, 98], and gene network dynamics. Regarding GRNs, a wide range of theoretical and empirical studies support that those networks with greater robustness to mutations are usually also robust to transient changes in gene activity, as those produced by developmental noise or environmental fluctuations [46, 99, 100]. Other simulation studies support that robustness to single-interaction mutations is also positively correlated to robustness to recombination [82] and to gene knock-outs [78]. Here we have extended these observations to robustness to gene duplication: we found a clear positive correlation between a GRN’s ability to buffer two kinds of perturbation: single-interaction mutation and gene duplication. The association of robustness to distinct sources of perturbation may be useful to comprehend its prevalence in biological systems [32, 101, 102]: evolution of robustness to some kind of perturbation may often have as a correlated effect the emergence of robustness to many other kinds of perturbations. Direct selection on robustness to genetic perturbations requires large population sizes and perturbation rates [103–106]. Nonetheless, since organisms are regularly subject to environmental fluctuations, developmental noise and other non-genetic disturbances, direct selection for robustness against such perturbations is feasible [44, 104, 107]. Robustness to gene duplication and other genetic perturbations may then appear gratuitously.

What is the effect of gene duplication on a GRN’s ability to buffer mutations? Our results suggest that the answer depends, to a large extent, on whether the duplication by itself produced phenotypic effects. When duplication implied a phenotypic alteration, mutational robustness decreased. However, those networks that preserved their original phenotype after duplication increased their ability to buffer mutations. This observation suggests that gene duplication can be partially responsible for the evolution of mutational robustness when networks are required to preserve the ability to yield an ancestral expression pattern. Research on GRN models has already established that networks with a greater number of genes can evolve more easily a greater mutational robustness [75]. Therefore, the effect that we observe after duplication on mutational robustness could be a mere consequence of the change in network size. However, this is not the case: we do not observe a positive effect on mutational robustness when a network acquires a new gene unrelated to other genes in the network. Hence, such an effect is specific for duplication.

Since the ability to maintain the ancestral phenotype after gene duplication seems an important distinction, we also asked what kind of genes are more likely to be duplicated without producing phenotypic effects. Neither how directly the duplicated gene regulated the structural genes that define a cell’s phenotype nor the number of genes that regulate its activity were consequential. In contrast, we found that regulator genes whose duplication bears no phenotypic effect tend to regulate fewer genes than duplicates that produce phenotypic alterations. This finding throws light on several previous observations. Work on model GRN’s had already shown that networks with a greater number of interactions display more severe phenotypic effects upon duplication than more sparsely connected GRNs [62]. In addition, computer simulations found that duplications of genes coding for proteins in the middle layers of signaling networks lack phenotypic effects more often than duplications of genes coding for proteins in the upper layers [63]. The authors argue that the reason may be that proteins in the top layers ultimately affect a greater number of genes. Moreover, it seems that non-essential genes are more prone to be retained as duplicates than essential genes and that such essential genes interact with more proteins [91]. All these observations support that genes that interact with a greater number of other genes have a lower probability to be retained in the genome as duplicates. A possible explanation, we contend, is that such promiscuous genes are more likely to produce deleterious phenotypic effects upon duplication.

The effect of duplication on buffering new mutations may not be the same for all kinds of single-interaction mutations. We hypothesized that whether a duplicate intervenes in the mutated interaction and whether mutation creates or deletes the interaction may be important factors. Indeed, that was the case. For mutations that delete an interaction, the effect is different depending on the regulator of the lost interaction. If such a regulator is a duplicate, it is more likely that the mutation has no phenotypic consequences. Whether the target in the missing interaction is a duplicate is of little relevance. The story is different for mutations that add a new interaction. In this case the nature of the regulator in the new interaction is nearly immaterial. However, the probability of maintaining the original phenotype is greater when the target in the new interaction is a duplicate. In this case, it makes little difference if the regulator is a duplicate or not.

Why do duplicates have different effects as targets and as regulators for interaction-adding and interaction-deleting mutations? A posteriori, the reason seems straightforward. Consider first interaction-deleting mutations. If a duplicate gene *D* regulates gene *X* and mutation deletes that interaction, the other copy $D'$ still regulates *X* in the same manner; that is, regulation of *X* returns to its condition before duplication. In contrast, if gene *Y* regulates the activity of two duplicates *D* and $D'$ and the regulation upon *D* disappears, then *D* might be active (or inactive) in conditions in which it usually is not, probably perturbing other network genes. Therefore, a duplicate losing the regulation of one of its targets is less often consequential than a duplicate losing one of its regulators.

Interaction-adding mutations do not follow the same pattern. If mutation creates a new interaction that regulates a duplicate *D*, *D* may present aberrant activity states. Nonetheless, the other duplicate $D'$ would still act as originally, counteracting some of *D*’s deviant effects. Alternatively, if the duplicate *D* is the regulator in the new interaction, nothing in $D'$ would obstruct transgressive regulation of other genes by *D*. Hence, phenotypic alterations are less likely when a duplicate acquires a new regulator than when it gains a new target.

We also studied how duplication affects a GRN’s ability to find new phenotypes through mutation. Congruently with our results on mutational robustness, we found that post-duplication mutational access to new phenotypes changes in a different manner for GRNs that preserve the original phenotype after duplication and for those that do not preserve it. After duplication, the number of phenotypes within reach of single mutations decreases for duplication-resistant GRNs but it increases for duplication-susceptible GRNs. When we check mutational access of new phenotypes after adding a new gene unrelated to other network genes, we note that it always increases. Thus, the effect that we observe after duplication is not merely due to the change in network size.

Gene duplication not only changes the number of phenotypes accessible through mutation. It also changes *which* phenotypes are accessible. Importantly, duplication-resistant GRNs after duplication retain mutational access to most of the phenotypes it could reach before duplication. GRNs that acquire a random non-duplicate gene do not. Importantly, those recurrently accessible phenotypes in duplication-resistant GRNs are more similar to the original phenotype. They also were the ones that, in the network before duplication, had a greater number of different mutations leading to them. That is, they were the ones that had easier mutational access. These observations suggest that gene duplication perturbs little a network’s ability to access the phenotypic variants that were within reach before duplication.

Taken together, our results support that duplication of regulator genes in gene regulatory networks often mitigates the impact of new mutations. Research on the relationship between mutational robustness and evolvability may help to reconcile this observation with the apparent role of gene duplication for adaptive evolution and innovation [1, 2, 18, 21, 22, 108]. An increase in mutational robustness allows a population to accumulate more cryptic genetic variation, which does not manifest phenotypically. This surplus genetic variation may eventually translate into a wider exploration of new phenotypic variants, increasing the chances of finding new beneficial variation [33–36].

Our work is consistent with a perspective in which gene duplication, even if not initially beneficial, allows biological systems to be better poised to respond to new evolutionary challenges. Hence, it is an expected result that evolution of metabolic capabilities almost from scratch occurs through adjustment of previously existing duplicates, many of them involved in transcriptional regulation [22]. In this perspective, retention of duplicates may be specially useful in populations that face frequent environmental changes, as shown in simulations of the evolution of GRNs under fluctuating environments [57] and also for invasive species [41]. Most of our observations are specific for duplication and not a mere consequence of change in network size. This is congruent with previous observations on simulations of the evolution of GRNs that suggest that network growth through addition of non-duplicate genes is not a significant factor for evolvability [109].

Too much additional experimental, theoretical and computational work is necessary for a better comprehension of the role of gene duplication in the evolution of gene regulatory networks. Here we focused on the early effects of gene duplication on the ability to produce a gene expression pattern when network dynamics start from a specific initial condition. Notwithstanding, in multicellular organisms, the network of gene interactions must be able to drive different cells from different initial conditions to distinct stable gene expression patterns. Addressing how duplication affects the different expression patterns that a network can produce is one of the many directions yet to follow.

## Supplementary information

**Additional file 1**. Illustration of the assessmentof similarity in terms of the Hamming distance between two phenotypes. Plus and minus signs refer to the activity state, active or inactive respectively, that the structural genes have at the end of a network’s dynamics. Different corresponding activity states in the two phenotypes appear in red. Phenotypes can be fixed points (*κ* = 1) or κ-period limit cycles (*κ* > 1). In (a) both phenotypes are fixed points. H(P1,P2) refers to the normalized Hamming distance between two phenotypes, P1 and P2. Namely, it is the fraction of different activity states in the two phenotypes. Similarity, S(P1,P2), equals 1 minus the normalized Hamming distance. In (b) P2 is a two-step limit cycle. We obtain the Hamming distance between P1 and each row in P2. Thus, S(P1,P2) equals 1 minus the average of the normalized Hamming distance between P1 and each row in P2.

**Additional file 2**. Average *S*_*µ*_ after mutations. Average *S*_*µ*_ after different kinds of mutations per kind of gene and kind of regulator-target interaction.

**Additional file 3**. Effect of regulator and target genes on phenotypic similarity. Type II two-way Anova analyses for the effect of regulator and target genes on phenotypic similarity *S*_*µ*_ after different kinds of mutations.

## Data Availability

All the code and scripts required to replicate our analyses are available at https://github.com/cespinosas/Duplication2022.
