# Supplementary material for: Early effects of gene duplication on the robustness and phenotypic variability of gene regulatory networks
Source: BMC Bioinformatics. 2022 Nov 28;23:509. doi: 10.1186/s12859-022-05067-1 (PMC9706961; doi:10.1186/s12859-022-05067-1)
Supplement: Supplementary file 1 — Additional file 1. Illustration of the assessmentof similarity in terms of the Hamming distance between two phenotypes. Plus and minus signs refer to the activity state, active or inactive respectively, that the structural genes have at the end of a network’s dynamics. Different corresponding activity states in the two phenotypes appear in red. Phenotypes can be fixed points (κ = 1) or κ-period limit cycles (κ > 1). In (a) both phenotypes are fixed points. H(P1,P2) refers to the normalized Hamming distance between two phenotypes, P1 and P2. Namely, it is the fraction of different activity states in the two phenotypes. Similarity, S(P1,P2), equals 1 minus the normalized Hamming distance. In (b) P2 is a two-step limit cycle. We obtain the Hamming distance between P1 and each row in P2. Thus, S(P1,P2) equals 1 minus the average of the normalized Hamming distance between P1 and each row in P2. [file 12859_2022_5067_MOESM1_ESM.pdf]

Illustration of the assessment of similarity in terms of the Hamming distance between two phenotypes.

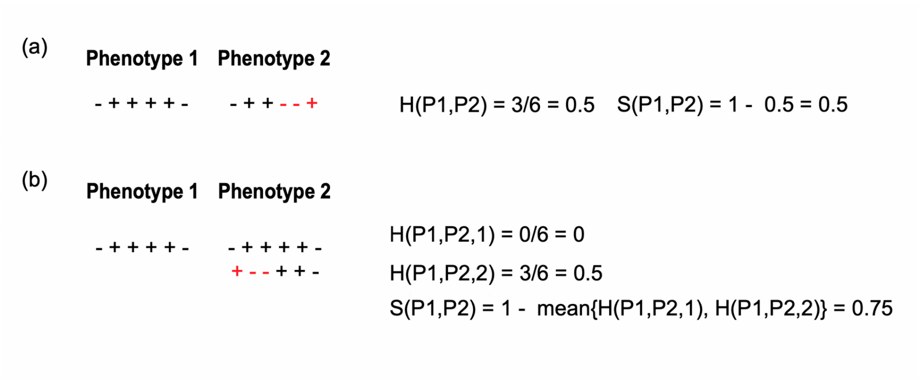

Plus and minus signs refer to the activity state, active or inactive respectively, that the structural genes have at the end of a network's dynamics. Different corresponding activity states in the two phenotypes appear in red. Phenotypes can be fixed points ($\kappa = 1$) or κ-period limit cycles ($\kappa > 1$). In (a) both phenotypes are fixed points. H(P1,P2) refers to the normalized Hamming distance between two phenotypes, P1 and P2. Namely, it is the fraction of different activity states in the two phenotypes. Similarity, S(P1,P2), equals 1 minus the normalized Hamming distance. In (b) P2 is a two-step limit cycle. We obtain the Hamming distance between P1 and each row in P2. Thus, S(P1,P2) equals 1 minus the average of the normalized Hamming distance between P1 and each row in P2.
