# Supplementary material for: Early effects of gene duplication on the robustness and phenotypic variability of gene regulatory networks
Source: BMC Bioinformatics. 2022 Nov 28;23:509. doi: 10.1186/s12859-022-05067-1 (PMC9706961; doi:10.1186/s12859-022-05067-1)
Supplement: Supplementary file 2 — Additional file 2. Average Sµ after mutations. Average Sµ after different kinds of mutations per kind of gene and kind of regulator-target interaction. [file 12859_2022_5067_MOESM2_ESM.pdf]

Average S_μ after mutations per kind of gene and kind of regulator-target interaction.

Factor	Deletion Mean±SD(n)	Addition Mean±SD(n)
**Regulator**		
D	$0.999 \pm 0.017(1,153)$	$0.951 \pm 0.049(1,631)$
N	$0.959 \pm 0.064(1,733)$	$0.955 \pm 0.048(1,764)$
**Target gene**		
D	$0.970 \pm 0.093(690)$	$0.965 \pm 0.064(1,043)$
N	$0.969 \pm 0.043(1,135)$	$0.926 \pm 0.044(1,176)$
S	$0.983 \pm 0.019(1,061)$	$0.969 \pm 0.014(1,176)$
**Regulator × Target gene**		
D→D	$0.997 \pm 0.036(133)$	$0.965 \pm 0.063(455)$
N→D	$0.964 \pm 0.101(557)$	$0.966 \pm 0.064(588)$
D→N	$0.998 \pm 0.165(547)$	$0.923 \pm 0.047(588)$
N→N	$0.942 \pm 0.043(588)$	$0.930 \pm 0.040(588)$
D→S	$> 0.999 \pm 0.005(473)$	$0.968 \pm 0.015(588)$
N→S	$0.970 \pm 0.017(588)$	$0.971 \pm 0.012(588)$
