# Supplementary material for: Early effects of gene duplication on the robustness and phenotypic variability of gene regulatory networks
Source: BMC Bioinformatics. 2022 Nov 28;23:509. doi: 10.1186/s12859-022-05067-1 (PMC9706961; doi:10.1186/s12859-022-05067-1)
Supplement: Supplementary file 3 — Additional file 3. Effect of regulator and target genes on phenotypic similarity. Type II two-way Anova analyses for the effect of regulator and target genes on phenotypic similarity Sµ after different kinds of mutations. [file 12859_2022_5067_MOESM3_ESM.pdf]

Type II two-way Anova analyses for the effect of regulator and target genes on phenotypic similarity S_μ after different kinds of mutations.

Factor(Df)	Deletion			Addition		
	$F(2880)$	p	η^2	$F(3389)$	p	η^2
Regulator(1)	445.72	$< 2 \times 10^{-16}$	0.130	5.44	0.0197	0.001
Target gene(2)	28.52	2×10^{-16}	0.017	330.02	$< 5.4\times10^{-13}$	0.163
Regulator × Target gene(2)	21.134	7.7×10^{-10}	0.012	0.95	0.3862	0.0004
